# Extreme phenotypes of the female athlete’s heart: a sports-specific cardiac magnetic resonance imaging study

**DOI:** 10.1093/ehjci/jeaf111

**Published:** 2025-06-02

**Authors:** Juliette C van Hattum, Maarten A van Diepen, Sjoerd M Verwijs, S Matthijs Boekholdt, Adrienne van Randen, Maarten Groenink, R Nils Planken, Maarten H Moen, Joëlle J N Daems, Niek H J Prakken, Danny A J P van de Sande, Birgitta K Velthuis, Suzanna de Vries, Ronald J Walhout, Yigal M Pinto, Arthur A M Wilde, Harald T Jørstad

**Affiliations:** Department of Cardiology, Amsterdam UMC Location University of Amsterdam, Room D3-221, Meibergdreef 9, 1105 AZ Amsterdam, The Netherlands; Amsterdam Cardiovascular Sciences - Heart Failure and Arrhythmias, University of Amsterdam, Meibergdreef 9, 1105 AZ Amsterdam, The Netherlands; Department of Cardiology, Amsterdam UMC Location University of Amsterdam, Room D3-221, Meibergdreef 9, 1105 AZ Amsterdam, The Netherlands; Amsterdam Cardiovascular Sciences - Heart Failure and Arrhythmias, University of Amsterdam, Meibergdreef 9, 1105 AZ Amsterdam, The Netherlands; Department of Cardiology, Amsterdam UMC Location University of Amsterdam, Room D3-221, Meibergdreef 9, 1105 AZ Amsterdam, The Netherlands; Amsterdam Cardiovascular Sciences - Heart Failure and Arrhythmias, University of Amsterdam, Meibergdreef 9, 1105 AZ Amsterdam, The Netherlands; Department of Cardiology, Amsterdam UMC Location University of Amsterdam, Room D3-221, Meibergdreef 9, 1105 AZ Amsterdam, The Netherlands; Amsterdam Cardiovascular Sciences - Heart Failure and Arrhythmias, University of Amsterdam, Meibergdreef 9, 1105 AZ Amsterdam, The Netherlands; Amsterdam Cardiovascular Sciences - Heart Failure and Arrhythmias, University of Amsterdam, Meibergdreef 9, 1105 AZ Amsterdam, The Netherlands; Department of Radiology and Nuclear Medicine, Amsterdam UMC Location University of Amsterdam, Amsterdam, The Netherlands; Department of Cardiology, Amsterdam UMC Location University of Amsterdam, Room D3-221, Meibergdreef 9, 1105 AZ Amsterdam, The Netherlands; Amsterdam Cardiovascular Sciences - Heart Failure and Arrhythmias, University of Amsterdam, Meibergdreef 9, 1105 AZ Amsterdam, The Netherlands; Amsterdam Cardiovascular Sciences - Heart Failure and Arrhythmias, University of Amsterdam, Meibergdreef 9, 1105 AZ Amsterdam, The Netherlands; Department of Radiology and Nuclear Medicine, Amsterdam UMC Location University of Amsterdam, Amsterdam, The Netherlands; High-Performance Team, Dutch National Olympic Committee and National Sports Federation, Arnhem, The Netherlands; Department of Cardiology, Amsterdam UMC Location University of Amsterdam, Room D3-221, Meibergdreef 9, 1105 AZ Amsterdam, The Netherlands; Amsterdam Cardiovascular Sciences - Heart Failure and Arrhythmias, University of Amsterdam, Meibergdreef 9, 1105 AZ Amsterdam, The Netherlands; Medical Imaging Center, Department of Radiology and Nuclear Medicine, University Medical Centre Groningen, University of Groningen, Groningen, The Netherlands; Department of Cardiology, UMC Utrecht, University of Utrecht, Utrecht, The Netherlands; Department of Radiology, UMC Utrecht, University of Utrecht, Utrecht, The Netherlands; Department of Cardiology, Tjongerschans Hospital, Heerenveen, The Netherlands; Department of Cardiology, Gelderse Vallei Hospital, Ede, The Netherlands; Department of Cardiology, Amsterdam UMC Location University of Amsterdam, Room D3-221, Meibergdreef 9, 1105 AZ Amsterdam, The Netherlands; Amsterdam Cardiovascular Sciences - Heart Failure and Arrhythmias, University of Amsterdam, Meibergdreef 9, 1105 AZ Amsterdam, The Netherlands; Department of Cardiology, Amsterdam UMC Location University of Amsterdam, Room D3-221, Meibergdreef 9, 1105 AZ Amsterdam, The Netherlands; Amsterdam Cardiovascular Sciences - Heart Failure and Arrhythmias, University of Amsterdam, Meibergdreef 9, 1105 AZ Amsterdam, The Netherlands; Department of Cardiology, Amsterdam UMC Location University of Amsterdam, Room D3-221, Meibergdreef 9, 1105 AZ Amsterdam, The Netherlands; Amsterdam Cardiovascular Sciences - Heart Failure and Arrhythmias, University of Amsterdam, Meibergdreef 9, 1105 AZ Amsterdam, The Netherlands; Amsterdam Movement Sciences, Vrije Universiteit, Amsterdam, The Netherlands

**Keywords:** female, athletes, ventricular remodelling, sports, MRI

## Abstract

**Aims:**

Differentiating physiological exercise-induced cardiac remodelling (EICR) from pathology is challenging, especially in female athletes, where studies using state-of-the-art imaging techniques are lacking. We aimed to investigate extreme phenotypes of EICR in female elite athletes using magnetic resonance imaging (MRI).

**Methods and results:**

Cross-sectional, multicentre study in female elite athletes using contrast-enhanced cardiac MRI. Left and right ventricle (LV, RV) indices and LV mass (LVM)-to-LV end-diastolic volume (EDV) ratios were investigated, indexed by body surface area (BSA). Cardiac remodelling was determined comparing cardiac MRI metrics to female reference values, stratified by ESC sports classification (endurance, mixed, power/skill). In 173 female elite athletes (median age 25 years, median 18 h training/week, 97% Caucasian), mean LV EDV/BSA and LVM/BSA were 108 ± 13 mL/m^2^ and 50 ± 10 g/m^2^, with lower LVM/LV EDV ratios (0.5 ± 0.1) than the general population (0.7 ± 0.1 g/mL; *P* < 0.001). Most athletes (71%) had isolated LV EDV increases; LVM increases (18%) commonly coincided with LV EDV increases. Compared with the general population (45 ± 7 g/m^2^), only mixed (48 ± 9 g/m^2^; *P* = 0.021) and endurance athletes (53 ± 11 g/m^2^; *P* < 0.001) exhibited greater mean LVM with endurance athletes surpassing mixed athletes (*P* = 0.004). Mixed and endurance showed comparably greater median biventricular EDV compared with power/skill athletes [LV: 109 (103–119) and 111 (101–118) vs. 99 (92–105); RV: 110 (103–118) and 112 (105–124) vs. 101 (95–104) mL/m^2^; *P* < 0.001]. Maximum wall thickness > 11 mm was rare (2%). Global T1 time was 968 ± 22 ms; extracellular volume was 25 ± 4%.

**Conclusion:**

Female elite athletes, particularly endurance-trained athletes, display EICR marked by increased ventricular volumes, without prominent increases in LVM or wall thickness.

**Trial Registration Number:**

NL9328

## Graphical Abstract

**Graphical Abstract:**
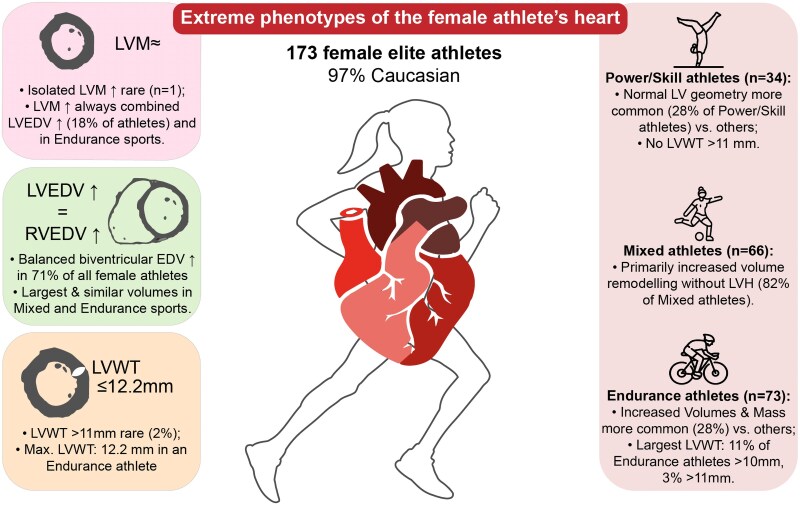
LV EDV, left ventricular end-diastolic volume; LVM, left ventricular mass; LVWT, left ventricular wall thickness; RV EDV, right ventricular end-diastolic volume.

## Introduction

Differentiation between physiological cardiac adaptation to sports and cardiac pathology remains a core challenge in sports cardiology.^1^ To aid this differentiation, clinicians often rely on reference ranges and indices for exercise-induced cardiac remodelling (EICR). However, current EICR reference ranges, indices, and the frameworks derived from these findings have been predominantly based on echocardiographic observations in male athletes.^2^

Data on EICR in female athletes is becoming increasingly pertinent, driven by the marked growth in the number of elite female athletes over recent decades, accompanied by a notable rise in training intensity and athletic performance.^3,4^ Although echocardiography has greatly advanced our understanding of sex-specific EICR, cardiac magnetic resonance imaging (cardiac MRI) is increasingly used and recognized as the non-invasive gold standard for detailed tissue characterization, also in athletes.^5,6^ Cardiac MRI more precisely quantifies cardiac indices, including the myocardium^7–9^ and right ventricular (RV) structure and function,^10^ further supplemented with techniques such as late gadolinium enhancement (LGE) and T1 and extracellular volume (ECV) mapping. At present, female athlete cardiac MRI-based, sports-specific EICR frameworks constitute a clear knowledge gap.^11^ Addressing this knowledge gap is important, as misinterpretation of findings in female athletes could lead to misdiagnoses, with serious consequences such as negative sports advice in the absence of true pathology, or false-negative interpretations of pathological findings.

Therefore, we aimed to investigate extreme EICR phenotypes using contrast-enhanced cardiac MRI in a large cohort of elite female athletes. We hypothesized that female elite athletes primarily demonstrate volumetric ventricular expansions and to a lesser degree increases in left ventricular (LV) myocardial mass and wall thickness, dependent on the type of sports.^12–14^

## Methods

### Study design

We performed a cross-sectional analysis of female elite athletes included in the prospective cohort of Evaluation of Lifetime participation in Intensive Top-level sports and Exercise (ELITE). The methods of ELITE have been described in detail elsewhere.^15^ In short, ELITE is an ongoing, prospective, multicentre longitudinal cohort study that collects standardized periodic cardiovascular data, including cardiac MRI, of elite athletes in The Netherlands. Athletes and the public are actively involved, as the Dutch National Olympic Committee & National Sports Federation is co-initiator of this study. ELITE received approval from the Amsterdam UMC Medical Ethics Committee (NL71682.018.19) and upholds principles of equity, diversity, and inclusion. The current analysis included cardiovascular screenings performed at the Amsterdam University Medical Centres, University Medical Centre Utrecht, Tjongerschans Hospital (Heerenveen), and Gelderse Vallei Hospital (Ede) between May 2019 and December 2024.

### Study population

We included female elite athletes (e.g. Olympic, national, and/or international athletes) of at least 16 years of age. Elite athletes were defined as athletes who perform at the highest national or international levels in sports and exercise more than 10 h per week with emphasis on competition and performance.^2^ All female athletes with a history of cardiovascular disease before cardiovascular screening and para-athletes were excluded. Athletes were approached by their personal or team/sports physician to voluntarily participate in this study; all included athletes provided written informed consent.

### Metrics of interest

Our primary metrics of interest were indices of EICR on cardiac MRI, expressed as LV remodelling index [LV wall mass (LVM) divided by LV end-diastolic volume (EDV)], compared with reference ranges for the general population.^16^ Additional metrics of interest included overall and sports-specific EICR for LV and RV EDV, LVM [indexed to body surface area (BSA)], and maximum LV wall thickness.

### Data collection

We collected clinical and cardiovascular screening data from included elite athletes. Demographics comprised age, sex, ethnicity, BSA, type of athletic discipline, total training hours per week, and years of participating on a professional athlete level. We recorded resting systolic (SBP) and diastolic blood pressure (DBP) and heart rate (HR) after supine resting electrocardiograms following a 5 min rest period. Additionally, if available, we collected cardiopulmonary exercise test (CPET) data, including VO₂ max, peak workload (Watt or METS), and HR, SBP, and DBP at peak exercise. CPETs were preferentially performed using a cycle ergometer or treadmill. We used unfiltered breath-by-breath gas analyses of O_2_ and CO_2_.

#### Cardiac MRI

Cardiac MRI was performed on 1.5 and 3.0 T MRI scanners (Siemens Avanto Fit 1.5T, Philips Ingenia 1.5T and Elition 3.0T), with a dedicated cardiac array RF coil for cardiac measurements. The scan protocol included cine imaging of long- and short-axis orientation, native modified look locker inversion recovery (MOLLI) T1 mapping in short-axis orientation, and LGE images (phase-sensitive inversion recovery), as previously described.^15^

Cardiac MRI analysis was performed in Circle Cardiovascular Imaging (cvi42 version 5.12.4, Calgary, Alberta, Canada) and Medis Medical Imaging (Medis Suite MR 4.0.62.4) by a dedicated core lab consisting of ELITE investigators (J.C.v.H., M.A.v.D., S.M.V., and J.J.N.D) and expert radiologists and imaging cardiologists (AT, B.K.V., MB, A.v.R., R.N.P., and M.G.) with experience in quantitative analyses of athlete cardiac MRI. Manually corrected artificial intelligence epi- and endocardial end-systolic and diastolic contours of both ventricles were used for all analyses. Short-axis cine images were used to determine LV and RV EDV and end-systolic volumes (ESV) (including papillary muscle and trabecularizations), stroke volumes (SV), ejection fractions (EF), and LV wall mass (LVM) (excluding papillary muscle and trabecularization). Volumes and LVM were indexed with BSA calculated by the Mosteller formula. Ratios of LV remodelling index (LVM/LV EDV) and left-right balance (LV EDV/RV EDV) were determined. The presence of LGE was determined on PSIR images through qualitative assessment and confirmed quantitatively if signal intensity exceeded six standard deviations above the normal myocardium.

T1 and ECV mapping data were exclusively used from 1.5 T scanners at Amsterdam UMC (Siemens Avanto Fit 1.5T) that have been validated in a previous study (Daems *et al*., under review). In short, global native T1 mapping and myocardial ECV calculations were performed using artefact-free, gadolinium-enhanced short-axis MOLLI sequences on three slices. ECV was derived using the following formula:^17^


$$\mathrm{ECV}=(1\text{–}\mathrm{haematocrit})\times (\Delta R1_{\mathrm{myocardium}}/\Delta R1_{\mathrm{blood}}),\;\mathrm{where}\;R1=1\;/\;T1$$

### Statistical methods

Categorical values are presented as numbers and percentages and continuous variables as mean and standard deviation (SD) or median with interquartile range (IQR), as appropriate. Normality of distribution was visually analysed with histograms and assessed using Shapiro–Wilk’s test. For our primary metrics of interest, we compared LV remodelling index (LVM/LV EDV) as well as other EICR metrics (means of biventricular EDV, ESV, systolic function, LVM, maximum LV wall thickness) of our study population to the general female population mean (papillary muscles included in ventricular volumes and excluded from LVM)^16^ with a one-sample *t*-test, expressed in percentage difference from reference mean. Furthermore, we used the BSA-indexed upper reference limit (URL) from the general female population^16^ to categorize EICR into four distinct groups: (i) normal geometry (both LVM and LV EDV within URLs); (ii) isolated LVM increase (LVM exceeding URL, LV EDV within URL); (iii) combined increases (both LVM and LV EDV exceeding URL); and (iv) isolated LV EDV increase (LV EDV exceeding URL, LVM within URL).

Sports were categorized according to the ESC sports classification (skill/power, endurance, or mixed), combining skill and power athletes due to their shared lack of endurance component and reliance on short bursts of intense exercise bouts, and to increase statistical power.^2^ We compared sports group-specific EICR to reference means and between groups using ANOVA, Kruskal–Wallis tests, *χ*² tests, or Fisher’s exact tests, as appropriate. Post hoc analyses included Tukey’s test following ANOVA or Dunn’s test following Kruskal–Wallis, both adjusted using Bonferroni correction. For individual sports with at least 10 athletes, cardiac MRI indices were also reported per sport.

We performed a backwards stepwise multiple linear regression analysis to explore the relationships between demographic and anthropometric variables, athlete characteristics (including ESC sports categories), and LV EDV, RV EDV, and LVM. Additionally, regression analysis was performed in a subgroup with available CPET data, using maximal work rate as an indicator of peak performance. The total variance explained by the multivariate models was quantified using adjusted *R*^2^.

Athletes with missing data for a specific variable were excluded from analyses pertaining to that variable and remained in all other analyses. The alpha level was set at 0.05. Statistical analyses are consistent with the CHAMP statement^18^ and were performed using R (version 4.4.2).

### Cardiac MRI interrater variability

Cardiac MRI intraclass correlation coefficients (ICC) were determined to assess the interrater variability between cardiac MRI analyses from the Amsterdam University Medical Centre and the University Medical Centre Utrecht. ICC estimates and their 95% confidence intervals (CIs) were calculated using a single-rating, absolute-agreement, two-way mixed-effects model.

## Results

### Population characteristics

We included a total of 173 female elite athletes between May 2019 and December 2024, with a median age of 25 (22–30) years, median BSA of 1.8 (1.7–1.9) m^2^, and a predominantly Caucasian ethnic background (97%) (*Table 1*). The athletes had performed median 12 (9–16) years on a professional athlete level with median 18 (18–20) sporting hours per week. Resting heart rate was 54 (47–60) bpm, at a resting SBP of 117 (111–123) and DBP of 70 (75–77) mmHg. In 79 athletes (46%), CPET data were available, with a median VO_2_ max of 47 (43–58) mL/kg/min. All athletes that underwent CPET demonstrated normal heart rate progression (peak 180 ± 12 bpm), with a normal blood pressure response [peak SBP 170 (160–180); peak DBP 70 (65–78) mmHg] at a peak work rate of 301 (274–334) Watts.

**Table 1:** Female athlete characteristics

Mean ± SD or median [Q1–Q3]	Overall
	(*n* = 173)
Age (years)	25 [22–30]
Mosteller BSA (m^2^)	1.8 [1.7–1.9]
**Ethnic class**	
Caucasian	168 (97%)
African/Afro-Caribbean	3 (2%)
East Asian and South Asian	1 (1%)
Latin American	1 (1%)
Sports participation (years)	12 [9–16]
Weekly sports (hours)	18 [18–20]
**Sports participation**	
Hockey	27 (16%)
Road cycling	27 (16%)
Water polo	16 (9%)
Rowing	13 (8%)
Soccer	11 (6%)
Artistic gymnastics	9 (5%)
Long track speed skating	9 (5%)
Sailing	9 (5%)
BMX racing	7 (4%)
Swimming	7 (4%)
Athletics	6 (4%)
Track cycling	6 (4%)
Miscellaneous^a^	26 (15%)
Resting HR (bpm)	54 [47–60]
Max HR (bpm)	180 ± 12
Resting SBP (mmHg)	117 [111–123]
Resting DBP (mmHg)	70 [66–75]
Max SBP (mmHg)	170 [160–180]
Max DBP (mmHg)	70 [65–78]
VO_2_ max (mL/kg/min)	47 [43–58]
Max work rate (W)	301 [274–334]

BSA, body surface area; HR, heart rate; DBP, diastolic blood pressure; SBP, systolic blood pressure; VO_2_ max, relative oxygen uptake.

^a^See Supplementary data online, *Table S1* for complete (miscellaneous) sports participation.

### Cardiac MRI indices

**Figure 1:**
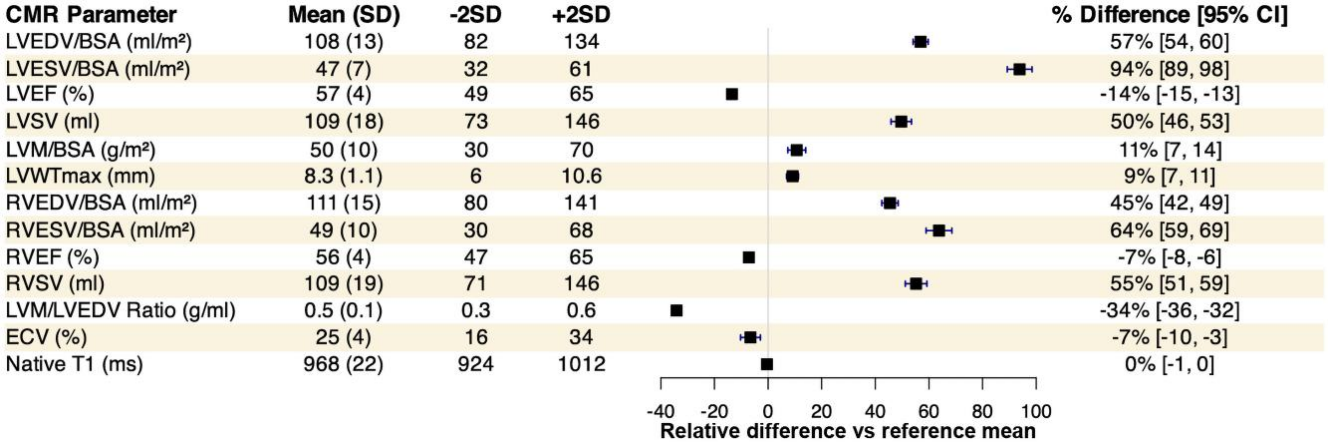
Forest plot depicting cardiac MRI indices, showing mean values with ±2 SD as reference and relative differences (in % and 95% CIs), comparing female elite athletes to the general female population. BSA, body surface area; ECV, extracellular volume; LV EDV, left ventricular end-diastolic volume; LV ESV, left ventricular end-systolic volume; LVSV, left ventricular stroke volume; LVEF, left ventricular ejection fraction; LVM, left ventricular wall mass; RV EDV, right ventricular end-diastolic volume; RV ESV, right ventricular end-systolic volume; RVSV, right ventricular stroke volume; RVEF, right ventricular ejection fraction; LVWTmax, left ventricular maximal wall thickness.

Overall, female elite athletes exhibited a mean LV EDV of 108 ± 13 mL/m^2^, RV EDV of 111 ± 15 mL/m^2^, LVM of 50 ± 10 g/m^2^, LV remodelling index of 0.5 ± 0.1 g/mL, and a mean maximum LV wall thickness of 8.3 ± 1.1 mm (*Figure 1*). Three athletes (2%) had an LV wall thickness exceeding 11 mm, with a maximum of 12.2 mm in an elite road cyclist (see Supplementary data online, *Figure S2*).

Compared with reference values from the general female population,^16^ female athletes demonstrated a 57% higher mean LV EDV/BSA (95% CI: 54–60% *P* < 0.001) and an 11% higher mean LVM/BSA (95% CI: 7–14%; *P* < 0.001). This resulted in 34% lower mean LV remodelling index (95% CI: −36 to −32%; *P* < 0.001). Athletes showed a 45% greater RV EDV/BSA (95% CI: 42–49%; *P* < 0.001) (*Figure 1*; also showing reference ranges mean −2 and +2SD).

Moreover, female athletes had markedly larger LV ESV/BSA at 47 ± 7 mL/m^2^, 94% higher than general population (95% CI: 89–98%; *P* < 0.001), and RV ESV/BSA at 49 ± 10 mL/m^2^, 64% higher (95% CI: 59–69%; *P* < 0.001). Consequently, athletes had mildly reduced ejection fractions, with LVEF at 57 ± 4% (14% lower; 95% CI: −15 to −13%; *P* < 0.001) and RVEF at 56 ± 4% (7% lower; 95% CI: −8 to −6%; *P* < 0.001) compared with the general population.

Gadolinium-enhanced images demonstrated 61 female athletes (35%) with inferior insertion point LGE, while one athlete (1%) displayed non-specific pericardial LGE without a history of symptomatic pericarditis. One athlete (1%) had non-specific focal LGE spots in multiple regions: basal anteroseptal, mid inferolateral, and mid anteroseptal with no clinical symptoms and no other cardiac abnormalities found during multi-modality cardiovascular work-up. Overall mean native T1 time was 968 ± 22 ms (*n* = 89), which was slightly, but not statistically significantly, lower than the non-sex-specific reference value (972 ± 43 ms; *P* = 0.071). ECV was slightly reduced at 25 ± 4% (*n* = 78), 7% lower than the general population (95% CI: −10 to −3%, *P* < 0.001), only one endurance athlete (1%) showed ECV below normal ranges at 19%.

ICC for reproducibility for ventricular volumes and mass on cardiac MRI ranged from 0.84 to 0.95, indicating good to excellent reliability (see Supplementary data online, *Table S2*).

#### Types of cardiac remodelling

**Figure 2:**
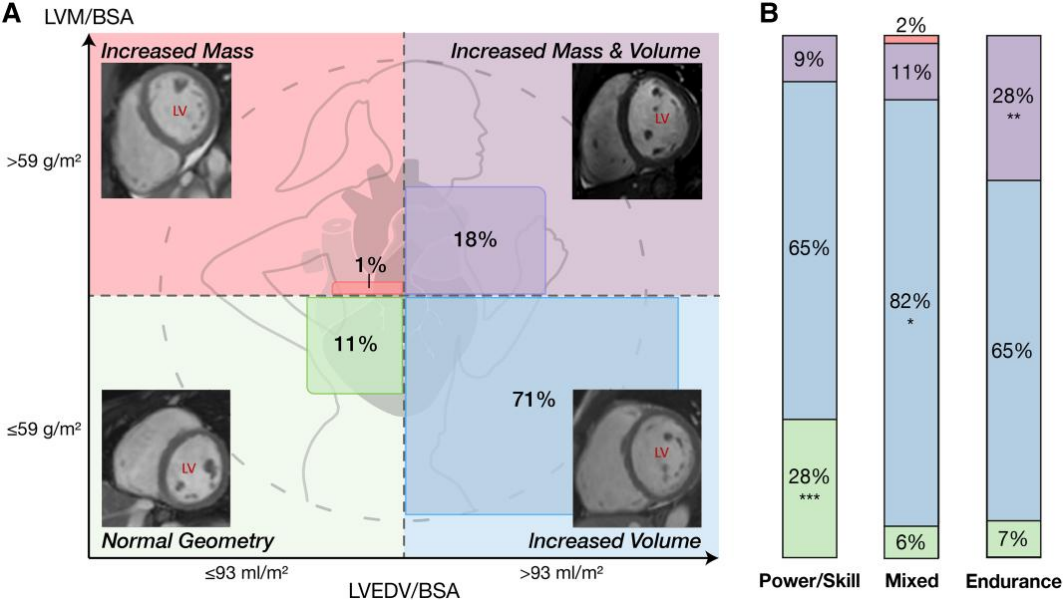
Distribution of extreme phenotypes of exercise-induced cardiac adaptation in female elite athletes (*n* = 173), (*A*) classified into quadrants based on the upper limits of reference values for left ventricular end-diastolic volume indexed to body surface area (LV EDV/BSA; 93 mL/m^2^) and left ventricular mass indexed to body surface area (LVM/BSA; 59 g/m^2^). (*B*) Bar plot illustrates the distribution of these phenotypes across sports categories: power/skill (*n* = 34), mixed (*n* = 66), and endurance (*n* = 73), with deviations from expected distributions indicated with asterisks (*P < 0.05, **P < 0.01, ***P < 0.001 from *χ*² test results). LV, left ventricle.

The most common EICR pattern was isolated increase in LV EDV/BSA with normal LVM/BSA, observed in 71% of athletes (*n* = 122) (*Figure 2*; Supplementary data online, *Figure S1*). This pattern was more frequent among mixed athletes (82%, *n* = 53, *P* < 0.05) compared with power/skill and endurance athletes (both 65%). The second most common pattern, seen in 18% of athletes (*n* = 30), was increased LV EDV/BSA and LVM/BSA. This was more prevalent in endurance athletes (28%, *n* = 20, *P* < 0.01) compared with power/skill (9%) and mixed athletes (11%). Normal cardiac geometry was observed in 11% of athletes (*n* = 18). Power/skill athletes were more likely to exhibit this pattern (26%, *n* = 9, *P* < 0.001) compared with mixed (6%) and endurance athletes (7%). Isolated increase in LVM/BSA was rare, occurring in only one athlete (1%), a field hockey player, with borderline increased LVM/BSA (59 g/m^2^) and borderline normal LV EDV/BSA (93 mL/m^2^).

#### Sports-specific findings

When stratified by ESC sports classification, the cohort consisted of 73 endurance athletes (42%), 66 mixed athletes (38%), and 34 power/skill athletes (20%) (*Table 2*; Supplementary data online, *Table S1*). Across all sports categories, athletes demonstrated higher mean LV and RV end-diastolic volumes compared with general female reference values (all *P* < 0.001). Only mixed (48 ± 8 g/m^2^, *P* = 0.020) and endurance athletes (53 ± 11 g/m^2^, *P* < 0.001) had higher mean LVM/BSA than the general population reference value (45 ± 7 g/m^2^).

**Table 2:** Sports-specific cardiac MRI parameters in female elite athletes

Mean ± SD or median [Q1–Q3]	Power/skill	Mixed	Endurance	*P*-value
	(*n* = 34)	(*n* = 66)	(*n* = 73)	
**Left ventricular**				
EDV/BSA (mL/m^2^)	99 [92–105]†‡	109 [103–119]*	111 [101–118]*	<0.001
ESV/BSA (mL/m^2^)	42 [39–46]†‡	46 [42–52]*	47 [43–52]*	0.005
SV (mL)	98 ± 17†‡	114 ± 18*	110 ± 17*	<0.001
EF (%)	57 ± 4	57 ± 4	57 ± 4	0.920
Mass/BSA (g/m^2^)	45 [41–50]‡	47 [42–50]‡	51 [46–60]*†	<0.001
WTmax (mm)	7.7 [7.2–9.1]	8.2 [7.5–8.9]	8.1 [7.7–9.1]	0.434
WTmax > 10 mm	1 (3%)	3 (5%)	8 (11%)	0.278
WTmax > 11 mm	0 (0%)	1 (2%)	2 (3%)	1.000
**LV geometry**				0.002
Increased mass and volume	3 (9%)	7 (11%)	20 (27%)	
Increased volume	22 (65%)	53 (80%)	47 (64%)	
Normal geometry	9 (27%)	4 (6%)	5 (7%)	
Increased Mass	0 (0%)	1 (2%)	0 (0%)	
**Right ventricular**				
EDV/BSA (mL/m^2^)	101 [95–104]†‡	110 [103–118]*	112 [105–124]*	<0.001
ESV/BSA (mL/m^2^)	44 [42–47]†‡	48 [43–56]*	50 [44–57]*	<0.001
SV (mL)	96 [86–105]†‡	113 [99–124]*	108 [97–118]*	<0.001
EF (%)	56 ± 4	56 ± 4	55 ± 5	0.296
**Tissue characterization**				
Global native T1 (ms)	970 ± 15	971 ± 22	964 ± 24	0.370
Global ECV (%)	26 [24–27]	25 [24–26]	24 [23–25]	0.299
**Ratios**				
LV mass to EDV ratio (g/mL)	0.5 [0.4–0.5]	0.4 [0.4–0.5]‡	0.5 [0.4–0.5]†	<0.001
LV to RV EDV ratio	1.0 ± 0.1	1.0 ± 0.1	1.0 ± 0.1	0.186

*: *P* < 0.05 vs. power/skill; †: *P* < 0.05 vs. mixed; ‡: *P* < 0.05 vs. endurance.

BSA, body surface area; ECV, extracellular volume; EDV, end-diastolic volume; EF, ejection fraction; ESV, end-systolic volume; LV, left ventricular; RV, right ventricular; SV, stroke volume; WTmax, maximum wall thickness.

**Figure 3:**
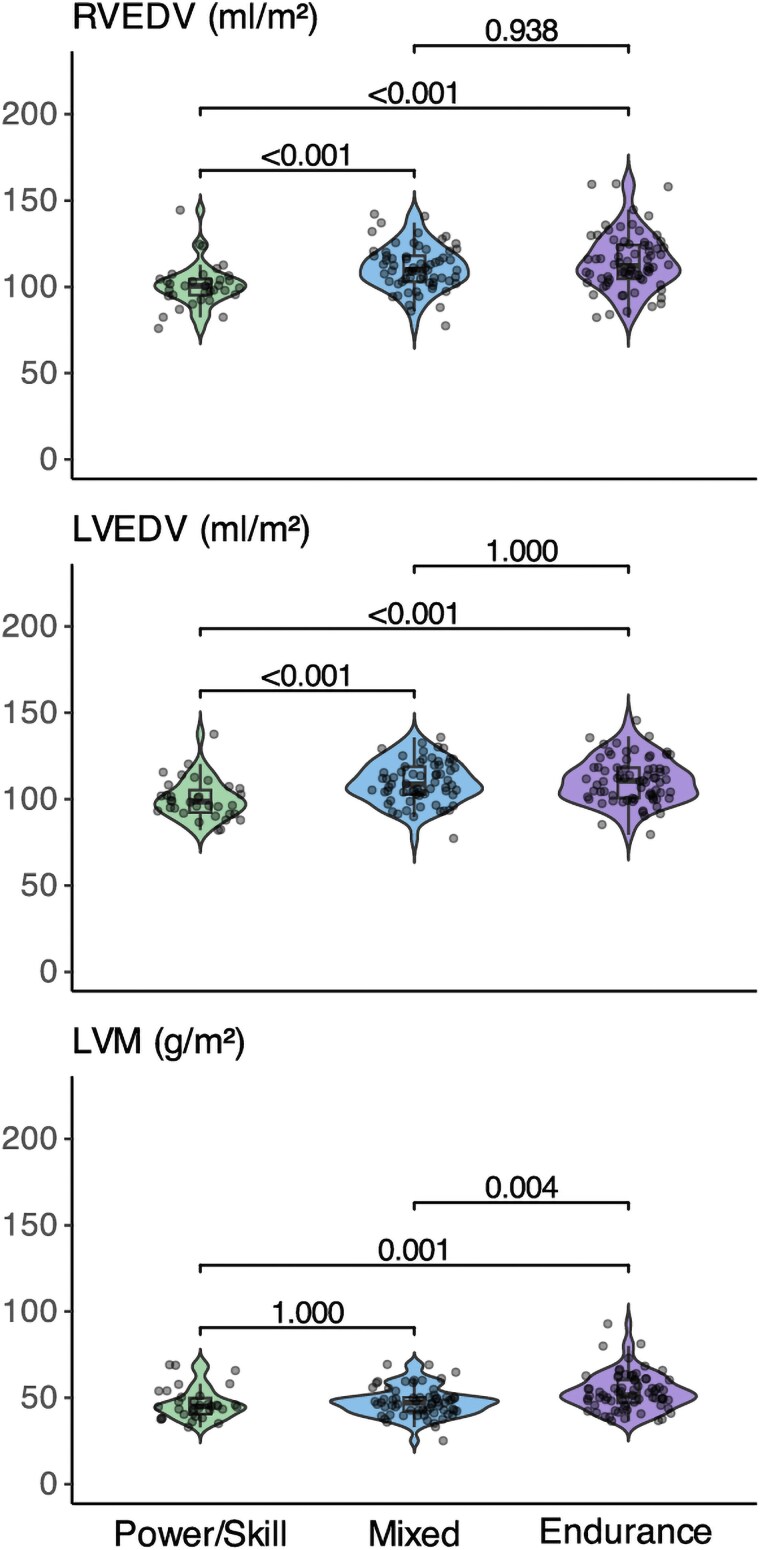
Violin plots illustrating sports-specific left and right ventricular adaptations in female elite athletes. *P*-values from Dunn’s post hoc results. LV EDV, left ventricular end-diastolic volume; LVM, left ventricular mass; RV EDV, right ventricular end-diastolic volume.

For both ventricles, median EDV was higher in endurance athletes [LV: 110 (101–118) mL/m^2^, *P* < 0.001; RV: 112 (105–124) mL/m^2^, *P* < 0.001] and mixed athletes [LV: 109 (103–119) mL/m^2^, *P* < 0.001; RV: 110 (103–118) mL/m^2^, *P* < 0.001] compared with power/skill athletes [LV: 99 (92–105) mL/m^2^, RV: 101 (95–104) mL/m^2^) (*Figure 3*). Median LVM was greater in endurance athletes [51 (46–60) g/m^2^] compared with power/skill [46 (41–50) g/m^2^, *P* = 0.001] and mixed athletes [47 (42–50) g/m^2^, *P* = 0.004]. LV remodelling index (LVM/LV EDV ratio) was, therefore, lower in mixed athletes compared with endurance athletes [0.4 (0.4–0.5) vs. 0.5 (0.4–0.5), *P* = <0.001), as the EDV was similar in mixed and endurance athletes (*P* = 0.938).

The median maximum LV wall thickness was similar across all groups [7.7 (7.2–9.1) to 8.2 (7.5–8.9) mm, *P* = 0.434]. There was a non-significant (*P* = 0.278) increase in the prevalence of maximum LV wall thickness > 10 mm: power/skill athletes (3%, *n* = 1), mixed athletes (5%, *n* = 3), and endurance athletes (11%, *n* = 8). Biventricular ejection fractions were consistent, with a similar LV ejection fraction at 57 ± 4% in all groups (*P* = 0.818) and RV ejection fractions ranging from 55 ± 5% in endurance athletes to 56 ± 4% in power/skill and mixed athletes (*P* = 0.296). LV-to-RV EDV ratios were balanced at 1.0 ± 0.1 in all groups (*P* = 0.186). Similarly, global mean native T1 time (964 ± 24 to 971 ± 22 ms, *P* = 0.370) and median ECV [24 (2325) to 26 (24–27), *P* = 0.299] showed no statistically significant variations between groups. However, endurance athletes had lower mean native T1 (964 ± 24 vs. 972 ± 43 ms; *P* = 0.049) and global ECV (24 ± 2% vs. 26 ± 3%; *P* < 0.001) compared with reference means. Supplementary data online, *Table S3* presents reference values for individual sports with at least 10 athletes.

### Linear regression analyses

In multivariate models, BSA strongly correlated with LV EDV (123 mL per m^2^), LVM (58 g per m^2^), and RV EDV (120 mL per m^2^; all *P* < 0.001) (*Table 3*). Resting heart rate demonstrated a negative association with all metrics (LV EDV: −0.7 mL per bpm; LVM: −0.7 g per bpm; RV EDV: −0.9 mL per bpm, all *P* < 0.001). Participation in endurance sports was independently associated with a differential 10 g greater LVM (*P* < 0.001), while participation in power/skill sports was independently correlated to 15 mL lower LV EDV (*P* = 0.002) and 18 mL lower RV EDV (*P* = 0.001).

**Table 3:** Multivariate linear regression analyses for LV EDV, LVM, and RV EDV

Variables	Coefficient	*P*-value
	**Multivariate LV EDV (mL)**	
BSA (per m^2^)	123	<0.001
Power/skill (yes)	−15	0.002
Resting HR (per bpm)	−0.7	<0.001
Adjusted *R*^2^	45%	
	**Multivariate LVM (g)**	
BSA (per m^2^)	58	<0.001
Endurance (yes)	10	<0.001
Resting HR (per bpm)	−0.7	<0.001
Adjusted *R*^2^	33%	
	**Multivariate RV EDV (mL)**	
BSA (per m^2^)	120	<0.001
Power/skill (yes)	−18	0.001
Resting HR (per bpm)	−0.9	<0.001
Adjusted *R*^2^	40%	

BSA, body surface area; HR, heart rate; LV EDV, left ventricular end-diastolic volume; LVM, left ventricular wall mass; RV EDV, right ventricular end-diastolic volume.

In athletes with available CPET data (see Supplementary data online, *Table S4*), peak work rate had positive associations in all multivariate models (LV EDV: 0.1 mL per W, *P* = 0.012; LVM 0.1 g per W, *P* = 0.001; RV EDV: 0.2 mL per W, *P* = 0.008). Inclusion of peak work rate negated the relationship of ESC sports categories with the ventricular metrics and the relationship between BSA and LVM (*P* = 0.057). The multivariate CPET models explained 41%, 35%, and 42% of the variance in LV EDV, LVM, and RV EDV, respectively.

## Discussion

This cardiac MRI study demonstrates that extreme cardiac phenotypes in elite female athletes predominantly manifest as balanced biventricular dilatation, with modest increases in cardiac mass and mildly reduced ECV. Isolated LV dilatation was most prevalent (71%) while concurrent increases in both LV EDV and LVM were seen in 18%, predominantly among endurance athletes. Furthermore, a maximal LV wall thickness of >11 mm was rare (2%), with only one endurance athlete slightly exceeding 12 mm. Athletes engaged in mixed and endurance exhibited more pronounced biventricular dilation, with increases in LVM observed almost exclusively in ‘pure’ endurance disciplines, and not in power/skill athletes, suggesting that endurance training induces specific cardiac adaptations in female athletes.

Our study emphasizes that volumetric expansion in the most common feature of female athlete EICR, rather than isolated mass increase. This finding of volumetric adaptation reinforces previous, primarily echocardiographic, studies.^14,19,20^ Unlike earlier echocardiographic studies that reported proportionally greater LV remodelling compared with the RV,^14^ we observed balanced biventricular sizes (LV/RV ratio of 1.0 ± 0.1). This discrepancy aligns with studies comparing both imaging modalities in female athletes^21^ and may be explained by the superior accuracy of cardiac MRI in quantifying RV morphology,^10^ thereby revealing an extent of volumetric RV remodelling beyond what echocardiography has detected. In addition, our exclusive focus on elite athletes—subjected to the highest training volumes and therefore likely to exhibit the most pronounced physiological adaptations—combined with LGE and tissue characteristics, confirms that the observed volumetric changes represent healthy adaptations rather than (subtle) pathology.^6^ This ‘extreme phenotype’ approach in the largest female contrast-enhanced cardiac MRI cohort to date aids in delineating the upper limits of physiological EICR. Consequently, our findings can assist clinicians in interpreting cardiac MRI results in female (elite) athletes, complementing earlier MRI studies that are limited by smaller sample sizes, varying sport classifications, and the use of non-contrast-enhanced protocols.^13,22,23^ Moreover, in contrast to our study, Maceira *et al*. reported greater LVM (73 g/m^2^) in a smaller (*n* = 52), selected sample of elite, female endurance athletes. Both our study and Luijkx *et al*.^13^—using similar MRI protocols in Dutch athletes—found lower LVM values (both 51 g/m^2^). These discrepancies could reflect population (Spanish vs. Dutch), or scanning protocol differences, highlighting the importance of larger-scale studies that both account for population heterogeneity and employ consistent imaging protocols with standardized athlete-specific myocardial quantification methods.

Of note, we found that an isolated increase of myocardial mass and thickness is uncommon in female athletes and occurs only in combination with pronounced ventricular dilatation. This is underscored by the mass-to-volume ratio of 0.5 g/mL, which is markedly lower than what is reported in the general female population (0.7 g/mL).^16^ We found one mixed athlete with an isolated, albeit borderline, LVM increase and one endurance athlete with a strong increase in LV wall thickness. No single power (or static) athlete demonstrated noteworthy LV hypertrophy. As such, the Morganroth hypothesis,^24^ which is largely based on echocardiographic data in male athletes, should be applied with caution and nuance in female athletes. This is of clinical importance, as next to ventricular dilatation, (concentric) hypertrophy is an anticipated finding in male power athletes^25^; its presence in female (non-endurance) athletes may however constitute a potential marker of cardiac disease, particularly in symptomatic individuals.^12,26^ Potential mechanisms for sex-specific differences in EICR include sex hormone levels,^27^ anthropometrics,^28^ and peak (exercise) blood pressure,^29^ but comparative studies are needed to further elucidate these differences.

Sports-specific EICR has been reported in both male and female athletes.^12,13,26^ Nevertheless, we identified a distinct EICR phenotype in female endurance athletes, marked by pronounced biventricular dilatation and increased myocardial mass compared with reference values and athletes from other disciplines. While mixed and endurance athletes exhibited similar biventricular volumes, only endurance athletes demonstrated noteworthy increases in LVM, leading to a relatively lower remodelling index in mixed athletes, which is consistent with previous studies.^13^ Our study reinforces the finding that prolonged endurance training in female athletes is associated with a distinct adaptation profile of eccentric LV hypertrophy, i.e. increased myocardial mass and volume.

### Clinical implications

This study assists clinicians in differentiating between physiological cardiac adaptation and potential cardiac pathology in female athletes. Our study provides specific female athlete cardiac MRI reference values and identifies volumetric enlargement as the primary hallmark of physiological cardiac adaptation to elite sports. Isolated, concentric hypertrophy or wall thickness exceeding 11 mm should be considered suspicious of cardiac pathology, especially in non-endurance sports, when evaluating Caucasian female athletes. Future international collaborations are required to prospectively investigate the long-term effects and cardiac safety of extreme levels of sports-related cardiac remodelling in female athletes with more diverse ethnicities.

### Strengths and limitations

There are several strengths to our study. First, the findings from the unique and well-defined ELITE cohort of female elite athletes competing at the highest international or national level provide insights into extreme EICR phenotypes. Second, the utilization of state-of-the-art imaging techniques, i.e. contrast-enhanced cardiac MRI, allows for more accurate assessments of cardiac parameters, especially for the right ventricle, myocardium, and tissue characteristics, which extends validity and applicability of our findings and better captures the nuanced and subtle EICR in women. Third, our study encompassed a wide array of sports, albeit with a lower proportion of skill and power athletes, which facilitates the development of reference ranges across various athletic disciplines. These sports were stratified using the ESC classification, which is commonly used by clinicians to provide sport-specific recommendations to their athlete patients.

Some aspects of our study warrant consideration. First, this study included only Dutch elite athletes and therefore lacks ethnic diversity. Hence, international collaborations are urgently needed to phenotype female elite athletes’ hearts for other ethnicities. Second, our cardiac MRI metrics of interest may have been influenced by some heterogeneities, due to differences in machine vendors and software. However, the excellent ICCs between the different study sites and the core lab analyses demonstrate only minor intra-observer differences for the central metrics of interest. Third, to facilitate generalizability, we used internationally accepted reference ranges for the general female population,^16^ but potentially, more distinct and subtle EICR phenotypes could be identified in future studies where female elite athletes are compared with body size-, age-, and ethnically matched non-athlete controls. However, this was beyond the scope of the current analysis. Fourth, cardiac MRI evaluation is still challenging due to artefacts, arrhythmias, and high inter-examiner variability. This is especially relevant in our comparisons of cardiac MRI reference values; however, current reference ranges are widely applied in international protocols for interpreting clinical findings.^30,31^ Finally, advanced quantifiable parameters, such as feature tracking strain analysis, were not assessed. These subtle metrics may offer additional insights into cardiac remodelling patterns and further refine comparisons with reference values. Future research should aim to incorporate these parameters.

## Conclusion

Female elite athletes demonstrate distinct patterns of EICR when compared with the general female population. This remodelling is mainly characterized by a balanced increase in ventricular volumes, with increases in ventricular mass limited to endurance-trained athletes. Clinically relevant changes in LV wall thickness or tissue characteristics were minimal or absent, especially in disciplines involving static cardiac demands. Our findings point towards distinct, female-specific features of EICR.

## Supplementary Material

jeaf111_Supplementary_Data

## Data Availability

The data underlying this article will be shared on reasonable request to the corresponding author.

## Patient involvement

Athletes and the public are actively involved, as the Dutch National Olympic Committee & National Sports Federation is a co-initiator of this study.
