# Supplementary material for: Extreme phenotypes of the female athlete’s heart: a sports-specific cardiac magnetic resonance imaging study
Source: Eur Heart J Cardiovasc Imaging. 2025 Jun 2;26(7):1199–207. doi: 10.1093/ehjci/jeaf111 (PMC12206574; doi:10.1093/ehjci/jeaf111)
Supplement: jeaf111_Supplementary_Data [file jeaf111_supplementary_data.docx]

**Supplementary Material**


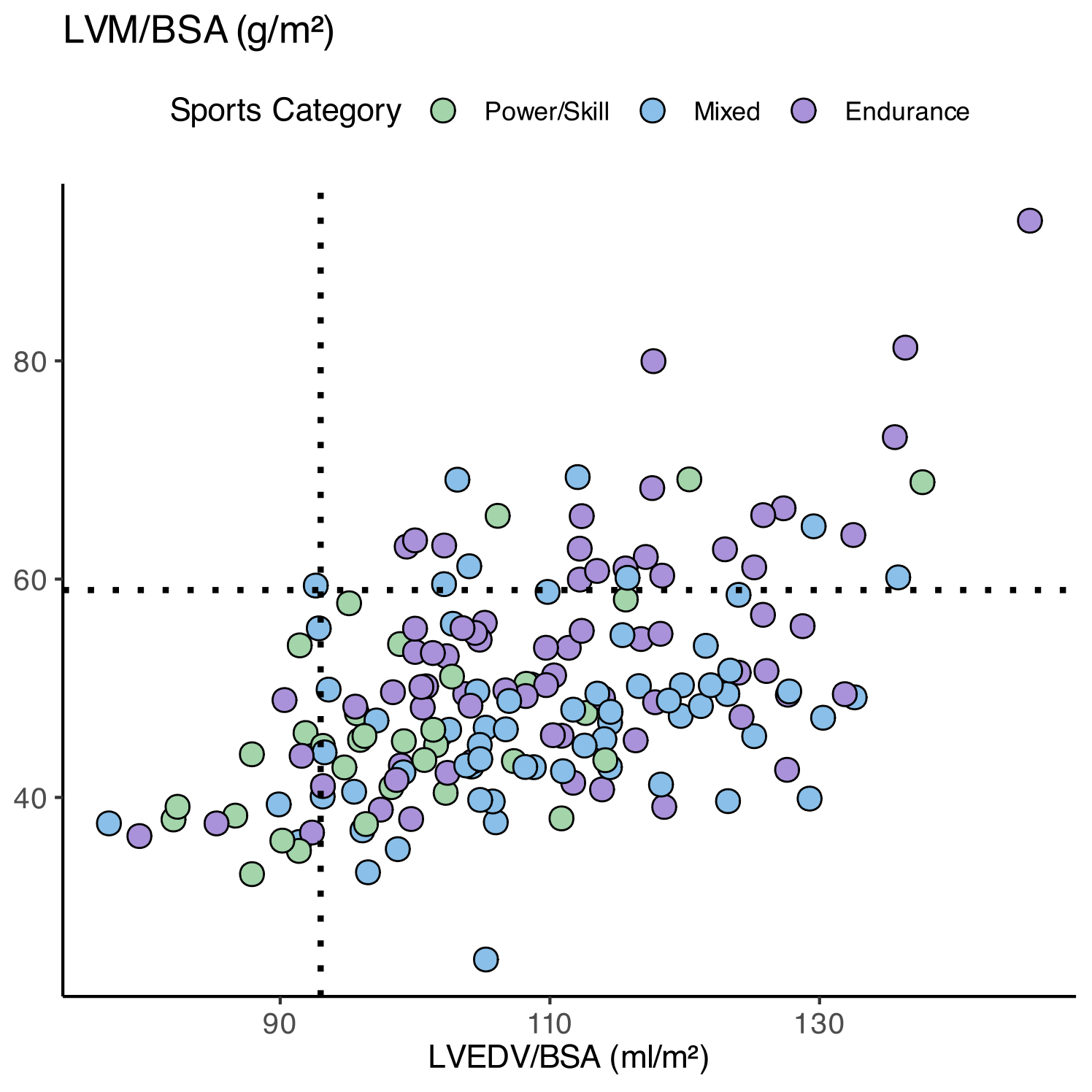


Supplementary Figure 1: Left ventricular remodeling patterns in female elite athletes. Each dot represents an athlete, color-coded by sports category. Dotted lines indicate upper reference limits based on general population reference values for left ventricular mass (LVM; 59 g/m^2^) and left ventricular end-diastolic volume (LVEDV; 94 ml/m^2^), indexed to body surface area (BSA).

**
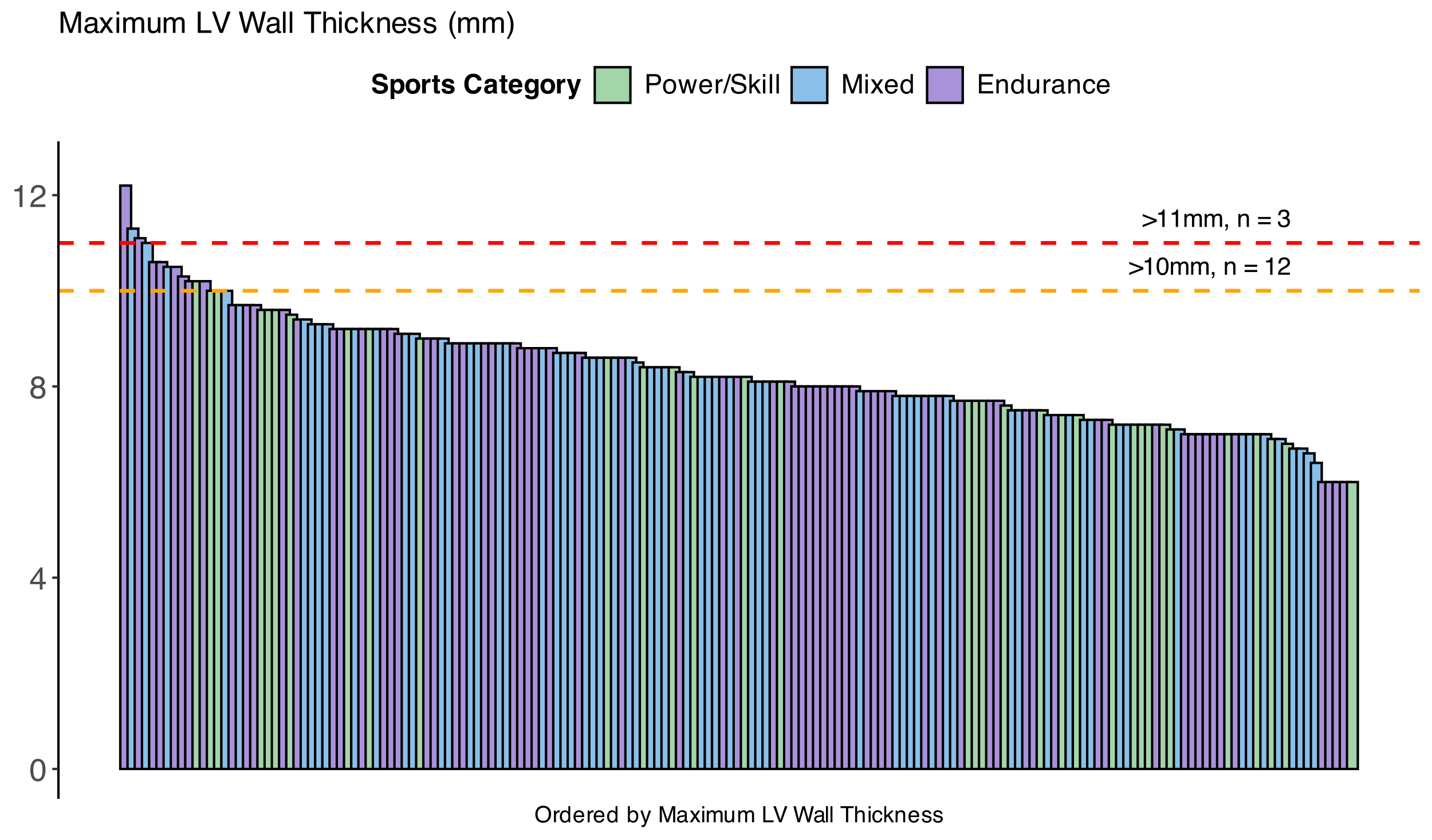
**

Supplementary Figure 2: Waterfall plot illustrating maximum left ventricular (LV) wall thicknesses ordered from high to low with dotted lines at as reference at maximum LV wall thickness of 10 and 11mm.

Supplementary Table 1: Female athlete characteristics per sports category

| *Mean ± SD / Median [Q1-Q3]* | **Power/Skill** | **Mixed** | **Endurance** | **P-value** |
| --- | --- | --- | --- | --- |
|  | **(n=34)** | **(n=66)** | **(n=73)** |  |
| Age (years) | 30 [23-31] | 25 [23-28] | 25 [22-28] | 0.041 |
| Mosteller BSA (m²) | 1.7 ± 0.1 | 1.8 ± 0.1 | 1.7 ± 0.1 | <0.001 |
| **Ethnic Class** |  |  |  | 0.161 |
| Caucasian | 31 (93.9%) | 64 (95.5%) | 72 (98.6%) |  |
| African/Afro-Caribbean | 2 (6.1%) | 1 (1.5%) | 0 (0%) |  |
| East Asian/South Asian | 0 (0%) | 0 (0%) | 1 (1.4%) |  |
| Latin American | 0 (0%) | 1 (1.5%) | 0 (0%) |  |
| Sports participation (years) | 16 [11–18] | 12 [10–15] | 10 [7–14] | 0.001 |
| Weekly sports (hours) | 18 [18–28] | 18 [13–18] | 19 [18–25] | <0.001 |
| **Sports participation** |  |  |  | - |
|  | Artistic gymnastics 9 (27%) | Basketball 2 (3%) | Artistic swimming 2 (3%) |  |
|  | Athletics 6 (18%) | Beach volleyball 2 (3%) | BMX racing 7 (10%) |  |
|  | Boxing 2 (6%) | Handball 3 (5%) | Long track speed skating 9 (12%) |  |
|  | Dressage 1 (3%) | Hockey 27 (41%) | Mountain biking 2 (3%) |  |
|  | Eventing 1 (3%) | Lacrosse 1 (2%) | Road cycling 27 (37%) |  |
|  | Judo 2 (6%) | Soccer 11 (17%) | Rowing 13 (18%) |  |
|  | Sailing 9 (27%) | Tennis 3 (5%) | Swimming 7 (10%) |  |
|  | Skateboarding 3 (9%) | Trampoline 1 (2%) | Track cycling 6 (8%) |  |
|  | Snowboard cross 1 (3%) | Water polo 16 (24%) |  |  |
| Resting HR (bpm) | 54 ± 10 (n = 33) | 55 ± 9 (n = 63) | 54 ± 10 (n = 71) | 0.909 |
| Max HR (bpm) | 174 ± 12 (n = 7) | 177 ± 9 (n = 25) | 183 ± 13 (n = 47) | 0.056 |
| Resting SBP (mmHg) | 112 [111 – 113] (n = 5) | 120 [114–130] (n = 33) | 115 [110–120] (n = 48) | 0.009 |
| Resting DBP (mmHg) | 72 [70 – 77] (n = 5) | 71 [68 – 78] (n = 33) | 70 [65–73] (n = 48) | 0.175 |
| Max SBP (mmHg) | 171 ± 17 (n = 6) | 172 ± 22 (n = 21) | 169 ± 13 (n = 36) | 0.791 |
| Max DBP (mmHg) | 70 [70–70] (n = 6) | 70 [55–70] (n = 21) | 75 [70 – 81] (n = 36) | 0.018 |
| VO_2_ Max (ml/kg/min) | 37 (n = 1) | 43 [40 – 46] (n = 18) | 55 [46 – 61] (n = 46) | <0.001 |
| Max Work Rate (W) | 285 [228 – 305] (n = 7) | 276 [250 – 301] (n = 23) | 325 [296–350] (n = 46) | <0.001 |

*Abbreviations: BSA = body surface area, HR = heart rate, DBP = diastolic blood pressure, SBP = systolic blood pressure, VO2 Max = Relative oxygen uptake*

Supplementary Table 2: Intraclass correlation coefficients of cardiac MRI parameters

|  | **Random rater ICC** | **95% CI** |
| --- | --- | --- |
| LVEDV | 0.98 | 0.91‐0.99 |
| LVESV | 0.91 | 0.44‐0.98 |
| LVM | 0.84 | 0.34‐0.94 |
| RVEDV | 0.94 | 0.80‐0.98 |
| RVESV | 0.84 | 0.43‐0.96 |
|  |  | *n=10* |

Supplementary Table 3: Individual sport-specific cardiac MRI indices for sports with ≥10 athletes, sorted by increasing means of left ventricular end-diastolic volume.

| *Mean (SD) / Median [Q1-Q3*] | **Soccer** | **Hockey** | **Water polo** | **Rowing** | **Road cycling** | **P-value** |
| --- | --- | --- | --- | --- | --- | --- |
|  | **(N=11)** | **(N=26)** | **(N=16)** | **(N=13)** | **(N=26)** |  |
| **Left Ventricular** |  |  |  |  |  |  |
| EDV (mL/m²) | 108 (11) | 110 (12) | 112 (15) | 113 (15) | 117 (11) | 0.168 |
| ESV (mL/m²) | 48 (6) | 47 (8) | 46 (6) | 49 (9) | 50 (8) | 0.695 |
| SV (mL) | 104 (17) | 110 (14) | 126 (21) | 122 (18) | 113 (14) | 0.003 |
| EF (%) | 56 (3) | 57 (4) | 58 (4) | 57 (4) | 58 (5) | 0.558 |
| Mass (g/m²) | 46 [38-48] | 47 [41-50] | 51 [47-59] | 54 [49-61] | 58 [52-63] | <0.001 |
| WTmax (mm) | 7.8 [7.0-8.4] | 8.5 [7.6-8.9] | 8.6 [8.1-9.0] | 8.9 [8.0-9.0] | 8.1 [7.8-9.5] | 0.083 |
| **Right Ventricular** |  |  |  |  |  |  |
| EDV (mL/m²) | 106 (10) | 111 (12) | 114 (17) | 118 (18) | 123 (15) | 0.011 |
| ESV (mL/m²) | 49 [43 - 50] | 46 [43 - 56] | 51 [40 - 57] | 54 [49 - 55] | 54 [48 - 64] | 0.200 |
| SV (mL) | 101 (15) | 109 (14) | 127 (21) | 123 (23) | 113 (14) | <0.001 |
| EF (%) | 55 (4) | 56 (4) | 58 (5) | 55 (5) | 55 (6) | 0.522 |
| **Tissue Characteristics** |  |  |  |  |  |  |
| Global native T1 (ms) | 969 (26) | 981 (19) | 950 (17) | 956 (16) | 958 (28) | 0.011 |
| Global ECV (%) | 25 [25 - 26] | 25 [23 - 27] | 25 [24 - 26] | 25 [24 - 26] | 25 [23 - 27] | 0.981 |
| **Ratios** |  |  |  |  |  |  |
| LV mass to EDV Ratio (g/mL) | 0.4 [0.4-0.4] | 0.4 [0.4-0.5] | 0.5 [0.4-0.5] | 0.5 [0.5-0.6] | 0.5 [0.5-0.5] | <0.001 |
| LV to RV EDV Ratio | 1.0 (0.1) | 1.0 (0.1) | 1.0 (0.1) | 1.0 (0.1) | 1.0 (0.1) | 0.169 |

*BSA = body surface area, ECV = extra cellular volume, EDV = end-diastolic volume, EF = ejection fraction, ESV = end-systolic volume, LV = left ventricular, RV = right ventricular SV = stroke volume, WTmax = maximum wall thickness*

Supplementary Table 4: Multivariate linear regression analyses for left ventricular end-diastolic volume (LVEDV) and mass (LVM), and right ventricular end-diastolic volume (RVEDV) that include CPET variables.

| **Variables** | **Coeff** | | ***P* value** |
| --- | --- | --- | --- |
|  | **Multivariate LVEDV (ml)** | | |
| BSA (per m2) | 113 | <0.001 | |
| Resting HR (per BPM) | -0.5 | 0.044 | |
| Maximal WR (per Watt) | 0.1 | 0.012 | |
| *Adjusted R^2^* | *41%* | | |
|  | **Multivariate LVM (g)** | | |
| BSA (per m^2^) | 30 | 0.057 | |
| Mixed (yes/no) | -8 | 0.108 | |
| Resting HR (per BPM) | -0.7 | <0.001 | |
| Maximal WR (per Watt) | 0.1 | 0.001 | |
| *Adjusted R^2^* | *35%* | | |
|  | **Multivariate RVEDV (ml)** | | |
| BSA (per m^2^) | 101 | <0.001 | |
| Endurance (yes) | 10 | 0.131 | |
| Resting HR (per BPM) | -0.9 | 0.003 | |
| Maximal WR (per Watt) | 0.2 | 0.008 | |
| *Adjusted R^2^* | *42%* | | |

*BSA=body surface area, HR=heart rate, LV EDV= left ventricular end-diastolic volume, LVM = left ventricular wall mass, RV EDV = right ventricular end-diastolic volume*
